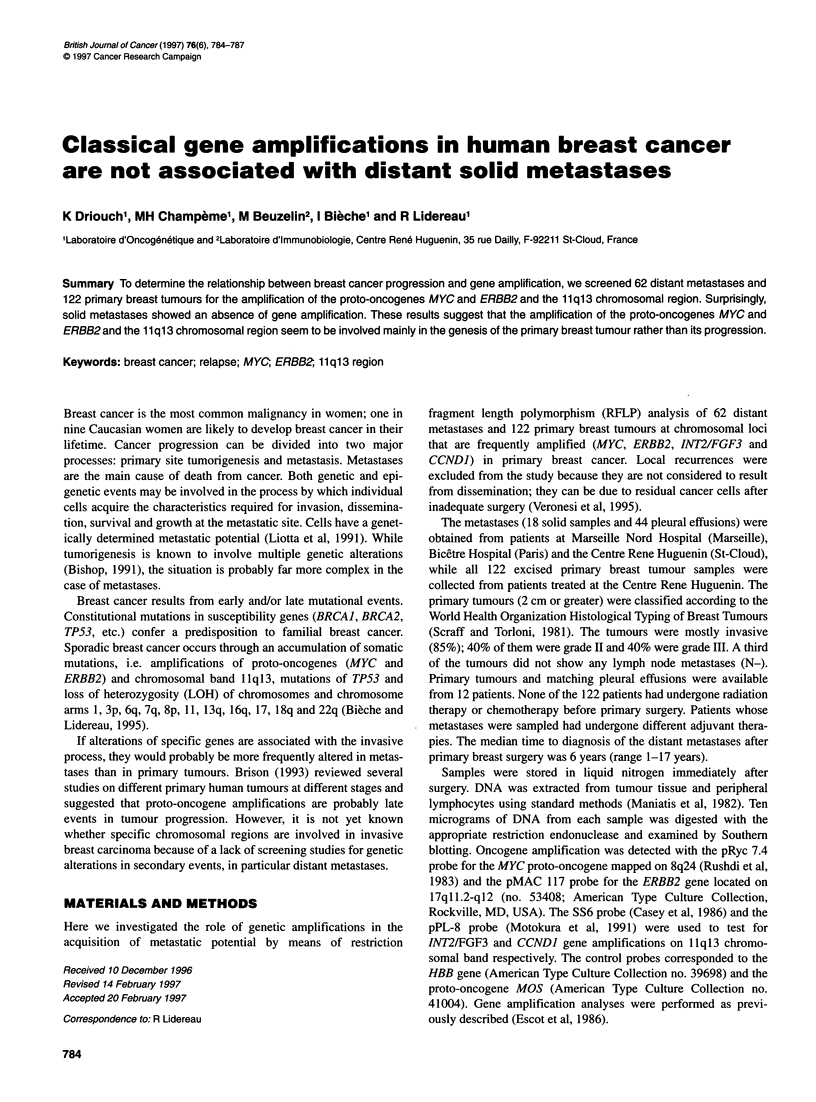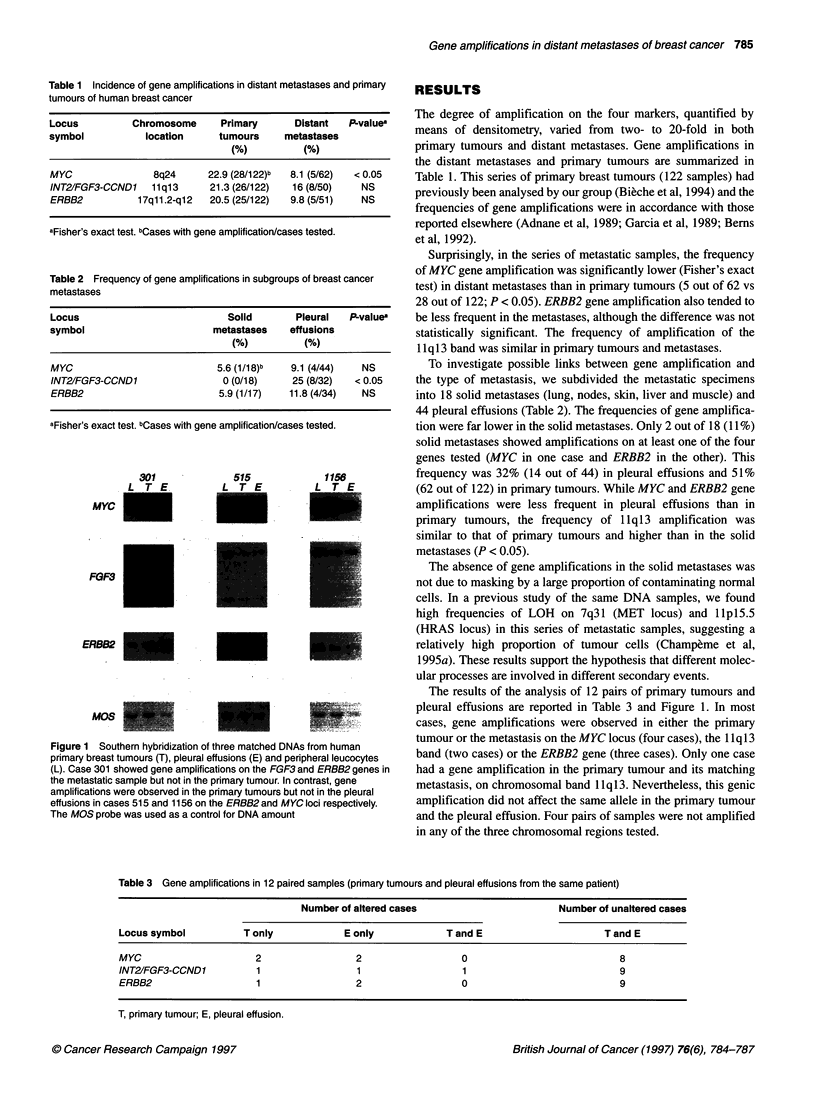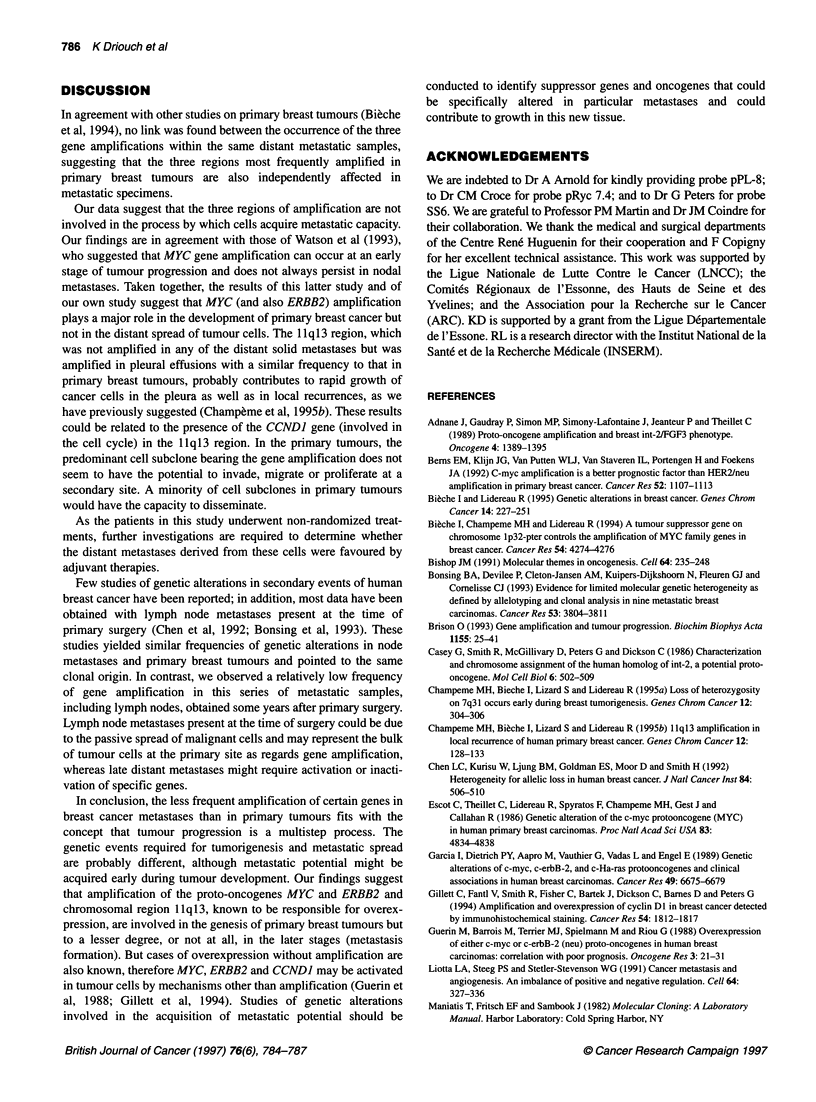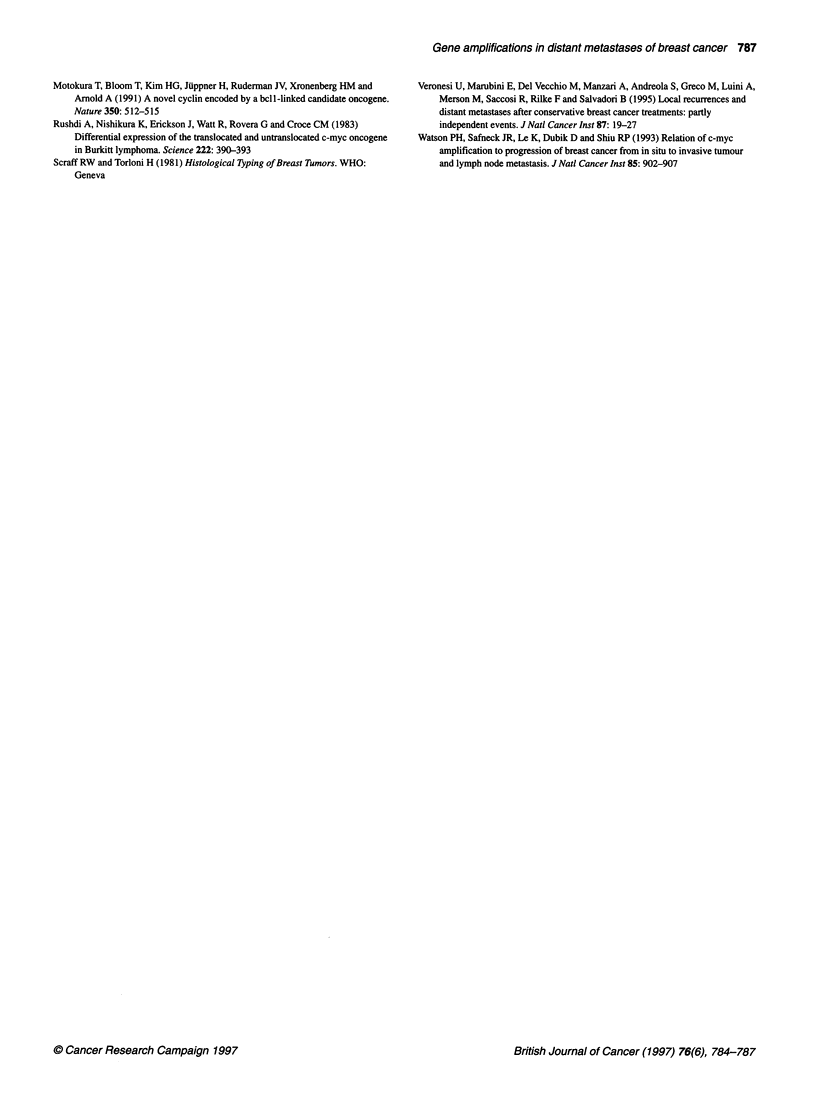# Classical gene amplifications in human breast cancer are not associated with distant solid metastases.

**DOI:** 10.1038/bjc.1997.462

**Published:** 1997

**Authors:** K. Driouch, M. H. ChampÃ¨me, M. Beuzelin, I. BiÃ¨che, R. Lidereau

**Affiliations:** Laboratoire d'OncogÃ©nÃ©tique, Centre RenÃ© Huguenin, St-Cloud, France.

## Abstract

**Images:**


British Journal of Cancer (1997) 76(6), 784–787
© 1997 Cancer Research Campaign

### Classical gene amplifications in human breast cancer are not associated with distant solid metastases

**K Driouch[1], MH Champème[1], M Beuzelin[2], I Bièche[1] and R Lidereau[1]**

[1]Laboratoire d'Oncogénétique and [2]Laboratoire d'Immunobiologie, Centre René Huguenin, 35 rue Dailly, F-92211 St-Cloud, France

**Summary** To determine the relationship between breast cancer progression and gene amplification, we screened 62 distant metastases and 122 primary breast tumours for the amplification of the proto-oncogenes *MYC* and *ERBB2* and the 11q13 chromosomal region. Surprisingly, solid metastases showed an absence of gene amplification. These results suggest that the amplification of the proto-oncogenes *MYC* and *ERBB2* and the 11q13 chromosomal region seem to be involved mainly in the genesis of the primary breast tumour rather than its progression.

**Keywords:** breast cancer; relapse; *MYC*; *ERBB2*; 11q13 region

Breast cancer is the most common malignancy in women; one in nine Caucasian women are likely to develop breast cancer in their lifetime. Cancer progression can be divided into two major processes: primary site tumorigenesis and metastasis. Metastases are the main cause of death from cancer. Both genetic and epigenetic events may be involved in the process by which individual cells acquire the characteristics required for invasion, dissemination, survival and growth at the metastatic site. Cells have a genetically determined metastatic potential (Liotta et al, 1991). While tumorigenesis is known to involve multiple genetic alterations (Bishop, 1991), the situation is probably far more complex in the case of metastases.

Breast cancer results from early and/or late mutational events. Constitutional mutations in susceptibility genes (*BRCA1*, *BRCA2*, *TP53*, etc.) confer a predisposition to familial breast cancer. Sporadic breast cancer occurs through an accumulation of somatic mutations, i.e. amplifications of proto-oncogenes (*MYC* and *ERBB2*) and chromosomal band 11q13, mutations of *TP53* and loss of heterozygosity (LOH) of chromosomes and chromosome arms 1, 3p, 6q, 7q, 8p, 11, 13q, 16q, 17, 18q and 22q (Bièche and Lidereau, 1995).

If alterations of specific genes are associated with the invasive process, they would probably be more frequently altered in metastases than in primary tumours. Brison (1993) reviewed several studies on different primary human tumours at different stages and suggested that proto-oncogene amplifications are probably late events in tumour progression. However, it is not yet known whether specific chromosomal regions are involved in invasive breast carcinoma because of a lack of screening studies for genetic alterations in secondary events, in particular distant metastases.

### MATERIALS AND METHODS

Here we investigated the role of genetic amplifications in the acquisition of metastatic potential by means of restriction fragment length polymorphism (RFLP) analysis of 62 distant metastases and 122 primary breast tumours at chromosomal loci that are frequently amplified (*MYC*, *ERBB2*, *INT2/FGF3* and *CCND1*) in primary breast cancer. Local recurrences were excluded from the study because they are not considered to result from dissemination; they can be due to residual cancer cells after inadequate surgery (Veronesi et al, 1995).

The metastases (18 solid samples and 44 pleural effusions) were obtained from patients at Marseille Nord Hospital (Marseille), Bicêtre Hospital (Paris) and the Centre Rene Huguenin (St-Cloud), while all 122 excised primary breast tumour samples were collected from patients treated at the Centre Rene Huguenin. The primary tumours (2 cm or greater) were classified according to the World Health Organization Histological Typing of Breast Tumours (Scraff and Torloni, 1981). The tumours were mostly invasive (85%); 40% of them were grade II and 40% were grade III. A third of the tumours did not show any lymph node metastases (N–). Primary tumours and matching pleural effusions were available from 12 patients. None of the 122 patients had undergone radiation therapy or chemotherapy before primary surgery. Patients whose metastases were sampled had undergone different adjuvant therapies. The median time to diagnosis of the distant metastases after primary breast surgery was 6 years (range 1–17 years).

Samples were stored in liquid nitrogen immediately after surgery. DNA was extracted from tumour tissue and peripheral lymphocytes using standard methods (Maniatis et al, 1982). Ten micrograms of DNA from each sample was digested with the appropriate restriction endonuclease and examined by Southern blotting. Oncogene amplification was detected with the pRyc 7.4 probe for the *MYC* proto-oncogene mapped on 8q24 (Rushdi et al, 1983) and the pMAC 117 probe for the *ERBB2* gene located on 17q11.2-q12 (no. 53408; American Type Culture Collection, Rockville, MD, USA). The SS6 probe (Casey et al, 1986) and the pPL-8 probe (Motokura et al, 1991) were used to test for *INT2*/FGF3 and *CCND1* gene amplifications on 11q13 chromosomal band respectively. The control probes corresponded to the *HBB* gene (American Type Culture Collection no. 39698) and the proto-oncogene *MOS* (American Type Culture Collection no. 41004). Gene amplification analyses were performed as previously described (Escot et al, 1986).

*Received 10 December 1996*
*Revised 14 February 1997*
*Accepted 20 February 1997*

*Correspondence to:* R Lidereau

**Table 1** Incidence of gene amplifications in distant metastases and primary tumours of human breast cancer

| Locus symbol | Chromosome location | Primary tumours (%) | Distant metastases (%) | *P*-value[a] |
|---|---|---|---|---|
| *MYC* | 8q24 | 22.9 (28/122)[b] | 8.1 (5/62) | < 0.05 |
| *INT2/FGF3-CCND1* | 11q13 | 21.3 (26/122) | 16 (8/50) | NS |
| *ERBB2* | 17q11.2-q12 | 20.5 (25/122) | 9.8 (5/51) | NS |

[a]Fisher's exact test. [b]Cases with gene amplification/cases tested.

**Table 2** Frequency of gene amplifications in subgroups of breast cancer metastases

| Locus symbol | Solid metastases (%) | Pleural effusions (%) | *P*-value[a] |
|---|---|---|---|
| *MYC* | 5.6 (1/18)[b] | 9.1 (4/44) | NS |
| *INT2/FGF3-CCND1* | 0 (0/18) | 25 (8/32) | < 0.05 |
| *ERBB2* | 5.9 (1/17) | 11.8 (4/34) | NS |

[a]Fisher's exact test. [b]Cases with gene amplification/cases tested.

**Figure 1** Southern hybridization of three matched DNAs from human primary breast tumours (T), pleural effusions (E) and peripheral leucocytes (L). Case 301 showed gene amplifications on the *FGF3* and *ERBB2* genes in the metastatic sample but not in the primary tumour. In contrast, gene amplifications were observed in the primary tumours but not in the pleural effusions in cases 515 and 1156 on the *ERBB2* and *MYC* loci respectively. The *MOS* probe was used as a control for DNA amount

### RESULTS

The degree of amplification on the four markers, quantified by means of densitometry, varied from two- to 20-fold in both primary tumours and distant metastases. Gene amplifications in the distant metastases and primary tumours are summarized in Table 1. This series of primary breast tumours (122 samples) had previously been analysed by our group (Bièche et al, 1994) and the frequencies of gene amplifications were in accordance with those reported elsewhere (Adnane et al, 1989; Garcia et al, 1989; Berns et al, 1992).

Surprisingly, in the series of metastatic samples, the frequency of *MYC* gene amplification was significantly lower (Fisher's exact test) in distant metastases than in primary tumours (5 out of 62 vs 28 out of 122; $P < 0.05$). *ERBB2* gene amplification also tended to be less frequent in the metastases, although the difference was not statistically significant. The frequency of amplification of the 11q13 band was similar in primary tumours and metastases.

To investigate possible links between gene amplification and the type of metastasis, we subdivided the metastatic specimens into 18 solid metastases (lung, nodes, skin, liver and muscle) and 44 pleural effusions (Table 2). The frequencies of gene amplification were far lower in the solid metastases. Only 2 out of 18 (11%) solid metastases showed amplifications on at least one of the four genes tested (*MYC* in one case and *ERBB2* in the other). This frequency was 32% (14 out of 44) in pleural effusions and 51% (62 out of 122) in primary tumours. While *MYC* and *ERBB2* gene amplifications were less frequent in pleural effusions than in primary tumours, the frequency of 11q13 amplification was similar to that of primary tumours and higher than in the solid metastases ($P < 0.05$).

The absence of gene amplifications in the solid metastases was not due to masking by a large proportion of contaminating normal cells. In a previous study of the same DNA samples, we found high frequencies of LOH on 7q31 (MET locus) and 11p15.5 (HRAS locus) in this series of metastatic samples, suggesting a relatively high proportion of tumour cells (Champème et al, 1995*a*). These results support the hypothesis that different molecular processes are involved in different secondary events.

The results of the analysis of 12 pairs of primary tumours and pleural effusions are reported in Table 3 and Figure 1. In most cases, gene amplifications were observed in either the primary tumour or the metastasis on the *MYC* locus (four cases), the 11q13 band (two cases) or the *ERBB2* gene (three cases). Only one case had a gene amplification in the primary tumour and its matching metastasis, on chromosomal band 11q13. Nevertheless, this genic amplification did not affect the same allele in the primary tumour and the pleural effusion. Four pairs of samples were not amplified in any of the three chromosomal regions tested.

**Table 3** Gene amplifications in 12 paired samples (primary tumours and pleural effusions from the same patient)

| | Number of altered cases | | | Number of unaltered cases |
|---|---|---|---|---|
| Locus symbol | T only | E only | T and E | T and E |
| *MYC* | 2 | 2 | 0 | 8 |
| *INT2/FGF3-CCND1* | 1 | 1 | 1 | 9 |
| *ERBB2* | 1 | 2 | 0 | 9 |

T, primary tumour; E, pleural effusion.

© Cancer Research Campaign 1997 

### DISCUSSION

In agreement with other studies on primary breast tumours (Bièche et al, 1994), no link was found between the occurrence of the three gene amplifications within the same distant metastatic samples, suggesting that the three regions most frequently amplified in primary breast tumours are also independently affected in metastatic specimens.

Our data suggest that the three regions of amplification are not involved in the process by which cells acquire metastatic capacity. Our findings are in agreement with those of Watson et al (1993), who suggested that *MYC* gene amplification can occur at an early stage of tumour progression and does not always persist in nodal metastases. Taken together, the results of this latter study and of our own study suggest that *MYC* (and also *ERBB2*) amplification plays a major role in the development of primary breast cancer but not in the distant spread of tumour cells. The 11q13 region, which was not amplified in any of the distant solid metastases but was amplified in pleural effusions with a similar frequency to that in primary breast tumours, probably contributes to rapid growth of cancer cells in the pleura as well as in local recurrences, as we have previously suggested (Champème et al, 1995*b*). These results could be related to the presence of the *CCND1* gene (involved in the cell cycle) in the 11q13 region. In the primary tumours, the predominant cell subclone bearing the gene amplification does not seem to have the potential to invade, migrate or proliferate at a secondary site. A minority of cell subclones in primary tumours would have the capacity to disseminate.

As the patients in this study underwent non-randomized treatments, further investigations are required to determine whether the distant metastases derived from these cells were favoured by adjuvant therapies.

Few studies of genetic alterations in secondary events of human breast cancer have been reported; in addition, most data have been obtained with lymph node metastases present at the time of primary surgery (Chen et al, 1992; Bonsing et al, 1993). These studies yielded similar frequencies of genetic alterations in node metastases and primary breast tumours and pointed to the same clonal origin. In contrast, we observed a relatively low frequency of gene amplification in this series of metastatic samples, including lymph nodes, obtained some years after primary surgery. Lymph node metastases present at the time of surgery could be due to the passive spread of malignant cells and may represent the bulk of tumour cells at the primary site as regards gene amplification, whereas late distant metastases might require activation or inactivation of specific genes.

In conclusion, the less frequent amplification of certain genes in breast cancer metastases than in primary tumours fits with the concept that tumour progression is a multistep process. The genetic events required for tumorigenesis and metastatic spread are probably different, although metastatic potential might be acquired early during tumour development. Our findings suggest that amplification of the proto-oncogenes *MYC* and *ERBB2* and chromosomal region 11q13, known to be responsible for overexpression, are involved in the genesis of primary breast tumours but to a lesser degree, or not at all, in the later stages (metastasis formation). But cases of overexpression without amplification are also known, therefore *MYC*, *ERBB2* and *CCND1* may be activated in tumour cells by mechanisms other than amplification (Guerin et al, 1988; Gillett et al, 1994). Studies of genetic alterations involved in the acquisition of metastatic potential should be conducted to identify suppressor genes and oncogenes that could be specifically altered in particular metastases and could contribute to growth in this new tissue.

### ACKNOWLEDGEMENTS

We are indebted to Dr A Arnold for kindly providing probe pPL-8; to Dr CM Croce for probe pRyc 7.4; and to Dr G Peters for probe SS6. We are grateful to Professor PM Martin and Dr JM Coindre for their collaboration. We thank the medical and surgical departments of the Centre René Huguenin for their cooperation and F Copigny for her excellent technical assistance. This work was supported by the Ligue Nationale de Lutte Contre le Cancer (LNCC); the Comités Régionaux de l'Essonne, des Hauts de Seine et des Yvelines; and the Association pour la Recherche sur le Cancer (ARC). KD is supported by a grant from the Ligue Départementale de l'Essone. RL is a research director with the Institut National de la Santé et de la Recherche Médicale (INSERM).

### REFERENCES

Adnane J, Gaudray P, Simon MP, Simony-Lafontaine J, Jeanteur P and Theillet C (1989) Proto-oncogene amplification and breast int-2/FGF3 phenotype. *Oncogene* **4**: 1389–1395

Berns EM, Klijn JG, Van Putten WLJ, Van Staveren IL, Portengen H and Foekens JA (1992) C-myc amplification is a better prognostic factor than HER2/neu amplification in primary breast cancer. *Cancer Res* **52**: 1107–1113

Bièche I and Lidereau R (1995) Genetic alterations in breast cancer. *Genes Chrom Cancer* **14**: 227–251

Bièche I, Champeme MH and Lidereau R (1994) A tumour suppressor gene on chromosome 1p32-pter controls the amplification of MYC family genes in breast cancer. *Cancer Res* **54**: 4274–4276

Bishop JM (1991) Molecular themes in oncogenesis. *Cell* **64**: 235–248

Bonsing BA, Devilee P, Cleton-Jansen AM, Kuipers-Dijkshoorn N, Fleuren GJ and Cornelisse CJ (1993) Evidence for limited molecular genetic heterogeneity as defined by allelotyping and clonal analysis in nine metastatic breast carcinomas. *Cancer Res* **53**: 3804–3811

Brison O (1993) Gene amplification and tumour progression. *Biochim Biophys Acta* **1155**: 25–41

Casey G, Smith R, McGillivary D, Peters G and Dickson C (1986) Characterization and chromosome assignment of the human homolog of int-2, a potential proto-oncogene. *Mol Cell Biol* **6**: 502–509

Champeme MH, Bieche I, Lizard S and Lidereau R (1995*a*) Loss of heterozygosity on 7q31 occurs early during breast tumorigenesis. *Genes Chrom Cancer* **12**: 304–306

Champeme MH, Bièche I, Lizard S and Lidereau R (1995*b*) 11q13 amplification in local recurrence of human primary breast cancer. *Genes Chrom Cancer* **12**: 128–133

Chen LC, Kurisu W, Ljung BM, Goldman ES, Moor D and Smith H (1992) Heterogeneity for allelic loss in human breast cancer. *J Natl Cancer Inst* **84**: 506–510

Escot C, Theillet C, Lidereau R, Spyratos F, Champeme MH, Gest J and Callahan R (1986) Genetic alteration of the c-myc protooncogene (MYC) in human primary breast carcinomas. *Proc Natl Acad Sci USA* **83**: 4834–4838

Garcia I, Dietrich PY, Aapro M, Vauthier G, Vadas L and Engel E (1989) Genetic alterations of c-myc, c-erbB-2, and c-Ha-ras protooncogenes and clinical associations in human breast carcinomas. *Cancer Res* **49**: 6675–6679

Gillett C, Fantl V, Smith R, Fisher C, Bartek J, Dickson C, Barnes D and Peters G (1994) Amplification and overexpression of cyclin D1 in breast cancer detected by immunohistochemical staining. *Cancer Res* **54**: 1812–1817

Guerin M, Barrois M, Terrier MJ, Spielmann M and Riou G (1988) Overexpression of either c-myc or c-erbB-2 (neu) proto-oncogenes in human breast carcinomas: correlation with poor prognosis. *Oncogene Res* **3**: 21–31

Liotta LA, Steeg PS and Stetler-Stevenson WG (1991) Cancer metastasis and angiogenesis. An imbalance of positive and negative regulation. *Cell* **64**: 327–336

Maniatis T, Fritsch EF and Sambook J (1982) *Molecular Cloning: A Laboratory Manual*. Harbor Laboratory: Cold Spring Harbor, NY

© Cancer Research Campaign 1997

Motokura T, Bloom T, Kim HG, Jüppner H, Ruderman JV, Xronenberg HM and Arnold A (1991) A novel cyclin encoded by a bcl1-linked candidate oncogene. *Nature* **350**: 512–515

Rushdi A, Nishikura K, Erickson J, Watt R, Rovera G and Croce CM (1983) Differential expression of the translocated and untranslocated c-myc oncogene in Burkitt lymphoma. *Science* **222**: 390–393

Scraff RW and Torloni H (1981) *Histological Typing of Breast Tumors*. WHO: Geneva

Veronesi U, Marubini E, Del Vecchio M, Manzari A, Andreola S, Greco M, Luini A, Merson M, Saccosi R, Rilke F and Salvadori B (1995) Local recurrences and distant metastases after conservative breast cancer treatments: partly independent events. *J Natl Cancer Inst* **87**: 19–27

Watson PH, Safneck JR, Le K, Dubik D and Shiu RP (1993) Relation of c-myc amplification to progression of breast cancer from in situ to invasive tumour and lymph node metastasis. *J Natl Cancer Inst* **85**: 902–907

© Cancer Research Campaign 1997